# Dental Fear and Associated Factors among Children and Adolescents: A School-Based Study in Lithuania

**DOI:** 10.3390/ijerph18168883

**Published:** 2021-08-23

**Authors:** Eglė Slabšinskienė, Aistė Kavaliauskienė, Miglė Žemaitienė, Ingrida Vasiliauskienė, Apolinaras Zaborskis

**Affiliations:** 1 Department of Oral Health and Paediatric Dentistry, Medical Academy, Lithuanian University of Health Sciences, LT-44307 Kaunas, Lithuania; migle.zemaitiene@lsmuni.lt (M.Ž.); ingrida.vasiliauskiene@lsmuni.lt (I.V.); 2 Department of Orthodontics, Faculty of Odontology, Medical Academy, Lithuanian University of Health Sciences, LT-44307 Kaunas, Lithuania; aiste.kavaliauskiene@lsmuni.lt; 3 Department of Preventive Medicine & Health Research Institute, Faculty of Public Health, Medical Academy, Lithuanian University of Health Sciences, LT-44307 Kaunas, Lithuania; apolinaras.zaborskis@lsmuni.lt

**Keywords:** oral health, dental fear, associated factors, children, adolescents, Lithuania

## Abstract

Dental fear is a challenging problem in dentistry and many contributing factors have been identified. Although this problem among children and adolescents has been studied in the literature for a long time, few such studies have been conducted in Lithuania. This study aimed to evaluate the prevalence of dental fear and examine its association with gender, age and several psychological and social factors among children and adolescents in Lithuania. The cross-sectional survey included a randomly selected sample (*n* = 1590) of children aged 11–14 and adolescents aged 15–18. The data were supplemented by interviewing the parents of these subjects (*n* = 1399). Dental fear was measured with a single five-score question. The data collection also included questions on oral health, socioeconomic status, oral health-related quality of life and self-esteem. Poisson regression analysis was used to assess the association between perception of dental fear and potential predictor variables. It was found that 32.2% (95% CI: 29.9–34.4%) of children and adolescents reported no fear of dental treatment, 12.5% (10.8–14.2%) of their peers were highly afraid of dental treatment, and other subjects assessed their dental fear gradually. Girls reported greater dental fear scores than boys, but the level of dental fear did not depend on the age. We identified the groups of subjects by gender and age, and a higher level of dental fear was significantly associated with untreated caries experience, a delay in the age of the subject’s first visit to the dentist, low self-esteem, low oral health-related quality of life, low overall life satisfaction and low family affluence. The results also suggested that dental fear could originate from previous toothache, dentists’ actions, high sensitivity in the child and poor psychological readiness for treatment. It was concluded that dental fear among Lithuanian children and adolescents is a common problem that is associated with gender and several dental, psychological and social factors. The findings indicate that school-based health policies, paediatric dentists and parents should be encouraged to focus on the psychosocial factors associated with dental fear because most of them can be prevented.

## 1. Introduction

Dental fear and anxiety (DFA) are common factors affecting oral health and clinical management among people of any age, but they appear to develop mostly in childhood and adolescence [1,2,3]. DFA in children and adolescents has been recognised in many countries as a great challenge for paediatric dentists [4,5]. Children’s dental fear seems to persist into adulthood and become chronic [6]. In addition, people suffering from DFA often have poorer oral health and oral health-related quality of life (OHRQoL) than their non-suffering counterparts [5,7,8]. Patients with severe DFA usually require more dental treatment time [9]. Therefore, the growing understanding and appreciation of the significance of DFA reflect its paramount importance in recent dentistry research, and preventing and intercepting DFA during young ages is considered a critical approach for improving oral health and dental experience [10].

The terms ‘dental fear’ and ‘dental anxiety’ refer to the negative feelings associated with dental treatment. They are almost identical; however, in the scientific literature, there is a slight difference in their meaning [5,9,11,12]. According to Klingberg and Broberg (2007), dental fear represents a normal emotional reaction to specific experiences acquired in the dentist’s office, whereas dental anxiety denotes a general state of apprehension that something dreadful is going to happen in relation to dental treatment [13]. Excessive dental anxiety is manifested by a phobia, which may result in prolonged avoidance of seeing a dentist and dental treatment [9]. Despite the differences observed, in epidemiological studies and dental practice, the terms ‘dental fear’ and ‘dental anxiety’ are often used interchangeably, referring to strong negative emotions associated with dental treatment among patients regardless of whether the specific criteria for the diagnosis of these states were met [1,3,4,11]. In light of this, we will give priority to the use of the term ‘dental fear’ against ‘dental anxiety’, as this term has also been used in the survey of the present study.

A number of indices have been developed to assess the severity of DFA in young patients used both in research and in everyday clinical practice [5,11,14]. Due to the variety of methods used, the literature reports diversity in the prevalence rates of DFA among children and adolescents, from 6% to 75%, as found by Grisolia et al. (2021) [5]. Notwithstanding this limitation, researchers are looking for factors that determine or are associated with the level and prevalence of dental fear. It is considered that DFA at a young age is a dynamic phenomenon and many contributing factors have been identified [4,6,9,15]. There is almost total agreement in the literature that girls and younger children are most often reported as more fearful than boys and older children [5,16,17]. Among other major factors associated with DFA in children and adolescents, previous negative dental experiences are often cited [5,6]. In this respect, the child’s first dental experience is of particular importance [18,19]. It was found that DFA is associated with oral health, especially with higher caries complications [20,21,22]. Moreover, fear of dental treatment can result in the formation of a vicious cycle leading to future delay of dental treatment, with a deterioration in oral health, which consequently reinforces the dental fear [23].

DFA are also associated with the psychosocial context, so it is important to characterise them in local or regional studies [24]. However, studies on the relationship between DFA and psychosocial factors are less numerous, especially in school-aged children. The recent meta-analysis by Alharbi et al. (2021) found minor associations between child dental anxiety and OHRQoL and limited associations between dental anxiety and self-esteem [25]. The effects of socioeconomic factors such as soceconomic family status and parental education on child dental fear have also been rarely studied [24,26,27]. Nevertheless, findings on these relationships were inconsistent or showed an inverse relationship [7,13,15,22]. Clearly, there is a need for further research on dental fear among children and adolescents, estimating its severity and revealing contributing factors in order to increase our understanding of the aetiology of the DFA syndrome [7,8,12,25,28]. Research findings in this direction clearly suggest that a better understanding of the factors causing dental fear and anxiety may help paediatric dentists to plan appropriate behaviour management and provide better treatment strategies [1,6,15].

In light of the presented findings, our study aimed to evaluate the prevalence of dental fear and examine its association with gender, age and several psychological and social factors among children and adolescents (aged 11 to 18 years) in Lithuania. Within this aim, we also sought to identify the antecedents of a child’s dental fear.

## 2. Materials and Methods

### 2.1. Study Design

The data on which this study is based were obtained as a part of the large population-based research project of the epidemiology of dental caries, malocclusion and OHRQoL among schoolchildren in Lithuania. The project followed the principles of the observational research with a cross-sectional design. A description of this project can be accessed for more detailed information [29,30].

The study conformed to the principles outlined in the World Medical Association Declaration of Helsinki. Ethical approval of the research project was granted by the Kaunas Regional Biomedical Research Ethics Committee (reference number BE-2-27). Surveys at schools were authorised by national, regional and school authorities. All the questionnaires were administered in paper form and anonymous. At all times, the data were processed according to Lithuanian data protection laws. The Strengthening the Reporting of Observational studies in Epidemiology (STROBE) guidelines were followed in reporting this study [31].

### 2.2. Sample and Data Collection

The target population was children and adolescents aged 11–18 years. According to the objectives of the main study, it was calculated that the initial sample should have 2000 subjects [29]. Such a sample size was recruited from 6th to 11th grade students using random cluster (school, class) sampling. Twenty-six randomly selected public schools from across Lithuania were asked to participate in the survey in the 2013/2014 school year.

Authorities of the selected schools were contacted by researchers and informed about all aspects of the study. Parents were then asked to provide written informed consent for their child’s participation in the survey, and parents of 1693 students (85% of the initial sample) provided consent. Simultaneously, parents were asked to complete a parental questionnaire, and parents of 1463 students (73% of the initial sample) completed questionnaires.

The self-completed student questionnaires were administrated in school classrooms by the class teacher. Only those students with the signed informed consent of their parents were asked to complete the questionnaire. Students did not know their parents’ answers when answering the questionnaire questions. Completed questionnaires were obtained from 1591 students (80% of the initial sample). Reasons for additional dropout were that students were absent on the day of the survey distribution, or that they declined to participate. Although an objective dental examination was also performed during the survey, only data from students’ and their parents’ questionnaires were used in this study. The cleaned and merged database of the present study consisted of 1590 student questionnaires and 1399 parental questionnaires.

### 2.3. Questionnaires

Two originally created self-reported questionnaires were used in this study. The first was the student (child) questionnaire, which consisted of questions assessing oral health as well as demographic, psychological and social issues.

*Fear about dental treatment* was an outcome (dependent) variable in this study. The students were questioned about their fear of the dentist using the ‘Dental Anxiety Question’, a specific single-item instrument taken from the study by Neverlien (1990) [32]. Their feelings about fear of dental treatment were rated on a 5-point scale with the following answer options: 1—not at all afraid; 2—a little afraid; 3—somewhat afraid; 4—very afraid; 5—terrified. Because there was no rational way to divide these options into two categories, such as ‘low fear’ and ‘high fear’, the original 5-point rating of dental fear was retained when analysing the data.

The remaining questions were employed to construct independent variables (predictors). Independent variables had to be no more than two or three categories to facilitate interpretation of the results in the analyses.

*Self-reported general health status.* This was categorised into two levels based on responses to the question ‘How would you describe your health?’ Responses of ‘excellent’, ‘good’ and ‘average’ were coded as the first category, ‘good health’, and the options ‘satisfactory’ and ‘bad’ were coded as the second category, ‘less good health’.

*Self-reported rating of caries experience.* Respondents were asked whether they had dental caries (tooth decay) or cavities to be treated by using three answer options: ‘Yes, I just noticed myself’, ‘Yes, the dentist said me the same’ and ‘No, not at all’. In the analyses, the first two options were combined into one category, ‘Treatment is needed’, which was compared with another category, ‘Not at all’.

*Self-reported rating of malocclusion.* The problem of malocclusion was clarified in an indirect way using the question ‘Have you ever noticed that your teeth are poor in shape, crowded or with large gaps, not eroded, or you have an irregular bite?’ Answer options were the same as above. In the analyses, the first two options were combined into one category, ‘Anomalies were observed’, which was compared with another category, ‘No anomalies’.

*Teeth brushing.* This was rated as ‘Regular’ if the respondent reported that they brushed their teeth with a brush and paste more often than once a day. Other answer options were considered as ‘Irregular’ tooth brushing.

*Self-esteem.* The Lithuanian version of Rosenberg’s Self-Image scale was used to measure self-esteem [33]. The scale consists of 10 statements with which the respondent can strongly disagree (none score), disagree (1 score), agree (2 scores) or strongly agree (3 scores). The scores of responses to all statements were summed (5 items were reversely formulated; thus, they were recoded to specify a single underlying construct). A higher sum score corresponded to higher self-esteem. Depending on the median value, the sum score was divided into two levels: 0–20 scores meant ‘low esteem’, 21–30 scores meant ‘high esteem’.

*Overall life satisfaction,* or global well-being, was rated using the ladder technique [33]. Children were asked to take a look at the drawn ladder, with steps numbered from zero (‘0’) at the bottom to ten (‘10’) at the top, with the instruction to suppose that the bottom of the ladder represents the worst possible life, and the top of the ladder represents the best possible life. Then, they were asked to indicate the step of the ladder at which they would place their life at present. Therein, the response was scored from 0 to 10 and was divided into two levels: 0–7 scores indicated ‘low life satisfaction’, 8–10 scores indicated ‘high life satisfaction’.

*Oral Health-Related Quality of Life (OHRQoL).* The Child Perception Questionnaire was used to evaluate OHRQoL. The Lithuanian version of this questionnaire was translated, revised and validated by Kavaliauskiene et al. [34]. The questionnaire is a 37-item instrument. The items are scored on a 5-point Likert scale ranging from 0 (‘never’) to 4 (‘every day or almost every day’). The scores for each item were added together to obtain a sum score of the whole instrument; higher sum scores referred to worse OHRQoL. In the present analysis, the sum score was divided into two levels based on the median value: 0–9 scores meant ‘high OHRQoL’, and 10 or more scores meant ‘low OHRQoL’.

*Family affluence.* This was measured by the Family Affluence Scale, which has been specially developed as a measure of the social position of young people [35]. The scale is simple and easy to answer, even for children. The present scale included four questions, which cover cars and home computer ownership, bedroom occupancy and travelling on holidays. A sum score was calculated for each respondent based on his responses to these four items and later categorised as ‘low affluence’ (0–3 scores); ‘medium affluence’ (4–5 scores) and ‘high affluence’ (6–7 scores).

*Urban/rural residence.* Based on the answers to the question ‘Where do you live?’, students were divided into two groups: living in ‘urban’ (answer option ‘town’) and living in ‘rural’ (answer options ‘small town’ or ‘village’) areas.

The second questionnaire was addressed to students’ parents. The information provided by the parent survey clarified children’s data in respect to their growing up.

*Age at the first child’s visit to the dentist.* Parental responses could range from 0 to 12 years, but in the analysis, the groups of children ‘up to 6 years’ and ‘6 years or older’ were gathered on the basis of median value.

*Parental opinion about child’s dental fear.* Parents were asked whether the child had a fear of dental treatment; suggested answer options were ‘has a dental fear’, ‘earlier had a dental fear’, ‘no dental fear’.

*Causes of dental fear.* In the case of having dental fear, parents were asked to provide one or more causes that may have contributed to the child’s dental fear. Parents were asked to think about these causes: too young a child, a toothache that occurred during the treatment, dentists’ actions, high sensitivity of the child, poor psychological preparation for a visit to the dentist.

### 2.4. Statistical Analysis

The analysis was performed using the SPSS statistical package (version 21; IBM SPSS Inc, Chicago, IL, USA). Descriptive statistics were first estimated as the means, standard deviations, medians, frequencies (n) and percentages (%). As appropriate, the 95% confidence interval (CI) for percentages was estimated by the bootstrapping option with 1000 number of samples. Pearson’s χ^2^ test of association was used to estimate the significance of the relationship between two categorical variables. Poisson’s regression analysis was undertaken in order to determine the extent to which each factor predicted the dental fear variable. The strength of the association between the dental fear variable and the predictor was assessed by the relative ratio of the fear score means, which shows how many times the mean of the dental fear score (outcome variable) increases as the predictor changes from the reference category to the contrast category [36,37,38]. The analysis was conducted in the entire sample by adjusting data for gender and age, as well as in stratified groups by gender adjusting data for age, and in three stratified groups by age adjusting data for gender. The group analysis was controlled assessing the interaction between demographic variables (gender and age) and the interaction between independent variables and demographic variables. Because the frequency of subjects in groups was uneven, the data were weighted to obtain an equal number (265 or 16.7%) of subjects in each of the six gender/age groups. *p*-values of <0.05 were taken to indicate the statistical significance of the associations examined.

## 3. Results

### 3.1. Sample Characteristics

Self-reported data on dental fear were collected from 1590 students: 663 (41.7%) boys and 927 (58.3%) girls. The mean age was 15.62 years (SD = 1.52). The respondents were distributed in three age groups. Table 1 shows the distribution of the sample by gender and age groups. The groups of 11–14-year-old boys and girls in comparison with other groups were relatively small, and girls older than 15 years were more likely to participate in this survey. Thus, in analyses, the data had to be weighted by gender and age group to achieve an equal number of subjects in each of the six groups by gender and age.

Table 2 displays the frequency of students’ reports by sample characteristics related to the study objectives. Most students (85.0%) rated their health as good, but many of them reported dental health problems arising from lesions of dental tissue or malocclusion. The groups of students by their socioeconomic and psychological characteristics are also presented, but the size of these groups is more relative than absolute due to the fact that the relationship between the presented characteristics and dental fear was studied.

In a similar way, Table 3 displays the frequency of reports by students’ parents. In this study, the questions answered by the parents were very important because they helped us to determine the age at which the child first visited the dentist and the parents’ opinions about why their child was afraid of dental treatment. According to a survey of parents, various causes of dental fear in children were possible, such as toothache, procedures performed by the dentist, the child being too young and others, but none of them had an exceptional frequency. Parents were also asked if the child had a fear of dental treatment, and 32.6% of positive responses were received. However, comparing the responses of parents and children, we found that disagreements were common. On the one hand, 12.8% of children reported that they were not at all afraid of dental treatment, even though their parents stated the opposite. On the other hand, 14.9% of the children admitted to being moderately or very afraid of dental treatment, but this was unexpected by their parents.

### 3.2. Level and Prevalence of Dental Fear

**Figure 1:**
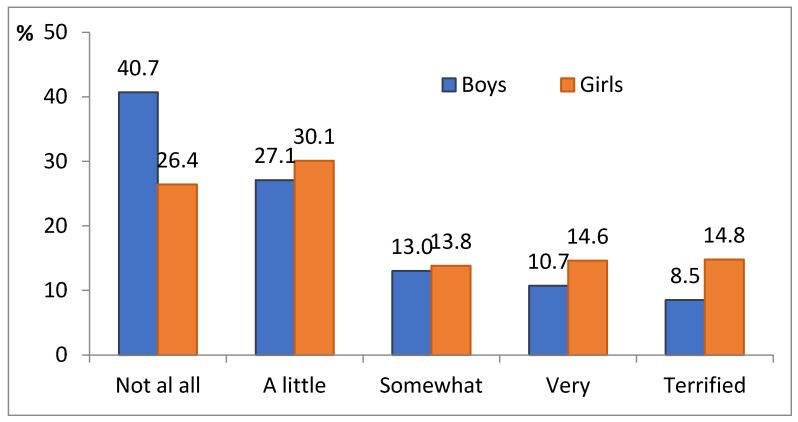
Response rates for boys and girls to the question about how much they were afraid of dental treatment (χ^2^ = 43.0; df = 4; *p* < 0.001).

In our survey, 32.2% (95% CI: 29.9–34.4%) of children and adolescents reported that they had absolutely no dental fear, while 12.5% (10.8–14.2%) of their peers said that they were highly afraid of dental treatment. Other students assessed dental fear gradually: a little—28.7% (26.5–30.8%); slightly—13.8% (12.2–15.5%); moderately—12.8% (11.1–14.5%). Scoring dental fear from 1 (no fear) to 5 (highly afraid), the mean score was 2.45 (95% CI: 2.38–2.52). This level may have changed due to various factors. For example, among boys and girls, the mean was 2.19 (2.10–2.28) and 2.62 (2.51–2.72), respectively, with a ratio of means of 1.20 (1.13–1.28), *p* < 0.001, which indicates that girls were more prone to dental fear. Figure 1 compares the response rates between boys and girls to the question of how much they were afraid of dental treatment.

### 3.3. Dental Fear and Its Association with Other Factors

Poisson regression helped us to determine the extent to which dental fear was associated with a variety of factors. The analysis was performed in the whole sample, as well as stratified by gender and age groups.

The data in Table 4 demonstrate how much the child and adolescent dental fear was associated with gender, age, self-reported health status and tooth brushing habits. As mentioned above, girls, regardless of their age, reported greater dental fear than boys. The level of dental fear did not significantly depend on the age of the respondents. The dental fear of any group of respondents had no relationship with the self-reported general health state, malocclusion or tooth brushing habits. One in three students reported having caries-damaged teeth that needed treatment. These respondents, except 11–14-year olds, felt a significantly higher level of dental fear compared with their peers with healthy teeth.

Table 5 shows an association of child and adolescent dental fear with several factors of psychological origin: self-esteem, oral health-related quality of life and overall life satisfaction. We can see that dental fear in both boys and girls was associated with low self-esteem, and this association was more pronounced among older adolescents. Similar association patterns were also found when examining the association of dental fear with low oral health-related quality of life; this association was significant in both boys and girls, and in 11–14-year-old children and 17–18-year-old adolescents. Low overall life satisfaction was also associated with higher levels of dental fear, but this factor was significant only among boys and the oldest adolescents.

Table 6 displays assessments of the association between child and adolescent dental fear and several social factors. Although there were no particularly significant associations here, some can be noticed. Higher levels of dental fear were more likely among girls living in rural areas, among girls from low-affluence families or among adolescents aged 17–18 years. Some significant relationships with parental education were also found but they emerged depending on the respondents’ age: a higher mean score of dental fear was found among 16–17-year-old adolescents whose mothers had lower education, and among 11–14-year-old children whose fathers had, on the contrary, lower education.

Table 7 provides results of analysis on the association of child and adolescent dental fear with variables reported by parents. The parents’ feeling that the child was afraid of dental treatment had a strong relationship with the child’s dental fear; the strength of this relationship depended neither on the child’s gender nor on their age. If the child’s first visit to the dentist was postponed to a later time (at 6 years or older), this was also negatively associated with dental fear, especially among 11–14-year-old children. Parents were asked to guess the reason for the child’s dental fear. The parental opinion that their child was too young during their first dental visit was not confirmed in any group of respondents. However, according to the parental opinion, it seems that children’s dental fear could have originated due to one or more of the following reasons: toothache that occurred during the treatment session, dentists’ actions, high sensitivity or poor psychological readiness for a visit to the dentist.

## 4. Discussion

This observational, cross-sectional, school-based study was focused on dental fear among children and adolescents (aged 11 to 18 years) in Lithuania. Most study participants were found to have reported high scores of dental fear, and girls reported greater dental fear than boys, but the level of dental fear did not depend on the age. Associated factors of dental fear were also investigated. It was found that untreated caries experience, a delay in the child’s first visit to the dentist, low self-esteem, low oral health-related quality of life, low overall life satisfaction and low family affluence were significantly associated with greater dental fear. The results also suggested that dental fear could originate from previous toothache, dentists’ actions, high sensitivity or poor psychological readiness for treatment, but not by the child being too young at his/her first visit to the dentist office.

The severity of dental fear was assessed using a single-item instrument taken from the study by Neverlien (1990) [32]. The high utility of such an approach was confirmed in such situations as national health surveys or in routine dental practice when the use of a longer dental anxiety/fear questionnaire is not feasible [20,32,39,40]. Moreover, multi-item self-reported scales have been criticised not only for the large number of questions and their complicated use in dental practice, but also for the lack of adequate explanation of the theoretical basis of the factors that they seek to measure [3,11]. The single question ensured that respondents had an opportunity to rate their own concern regarding how much they were afraid of dental treatment within a five-point score. The psychometric properties of this instrument, as evaluated in population-based studies, are considered adequate [15,32].

In our study, it was found that nearly one third (32.2%) of children and adolescents had absolutely no dental fear, and the remaining two thirds (67.8%) of respondents reported having more or less dental fear. These figures can be compared to other studies with a similar single question assessing dental fear. For example, in a Brazilian study by Colares et al. (2013), where dental fear was assessed in children aged 5 to 12 years old, a prevalence of 39.4% was observed [41]. In another study in Brazil, the occurrence of dental fear among 8- to-12-year-old children found a prevalence of 24.6% [42,43]. Olak et al. (2013) measured dental fear in 8- to-10-year-old children in Estonia with a single question and found a low prevalence (6.1%) of dental fear [44]. In general, a recent systematic review with meta-analyses conducted by Grisolia et al. (2021) reported that estimates of DFA prevalence in children and adolescents taken from 50 studies worldwide, which used different instruments, ranging from 6% to 75%, had a pooled prevalence of 23.9% (95% CI 20.4–27.3) [5]. The prevalence of DFA varies considerably across countries, with lower estimations in European children and higher estimations in Asian children [16,45]. Therefore, findings from our study in comparison with other studies indicate a relatively high severity of dental fear among the Lithuanian population aged 13–18 years. This conclusion is further supported by the fact that the sample that we examined was older than those in the studies cited above. There is almost total agreement in the literature that younger children tend to admit their fears more freely than older ones, and dental anxiety is expected to decrease as age advances [5,16,17]. The high prevalence (51.7%) of dental fear among Lithuanian children, aged 12–15, was also found in a study conducted by Račienė a decade ago [46].

The high prevalence of dental fear found in children and adolescents in Lithuania can have several explanations. On the one hand, this may be related to the high prevalence of caries and malocclusion among Lithuanian children and adolescents [29,30]. Therefore, most Lithuanian patients from childhood suffer from pain caused by caries or chewing disorders. Once these symptoms have been exacerbated, treating them can increase the negative experience, which reinforces the fear of visits to dentists or their procedures. An association of dental fear with the perception of pain during dental treatment has been found in many studies [5,6]. On the other hand, there is the possibility that dental fear behaviour may also be learned without direct experience [47,48]. For a long time, dental technology in Lithuania was lagged behind the advanced European level, so dental treatment was a very painful procedure, not only for children but also for adults [46,49]. The negative image of the dentist’s office has shaped anxiety about dental treatment, and such a phenomenon may still be widespread among the country’s population.

The next objective of this study was to test the association between dental fear and different groups of factors. From a methodological point of view, it was important to choose an appropriate method for assessing the association between variables [36,38]. In our study, the Poisson regression was chosen for this purpose. Such a choice was made for several reasons. Firstly, the distribution of respondents’ answers to the question about dental fear was close to the Poisson distribution. Secondly, the best analytic approach is to evaluate the association between symptoms and outcomes using the outcome as a continuous rather than a dichotomous variable [36]. Indeed, the diagnostic criteria of high and low dental fear have not been well specified and the necessary research has not been done [11,15,32]. The Poisson regression method satisfies all these requirements.

As there is still some debate on whether dental fear is associated with demographic variables [5], we tested whether dental fear varies by respondent gender and age. In all three age groups of our sample, the girls were found to be more fearful than boys, which is in line with most other studies [4,5,6,16,46]. In the literature, a usual explanation for this association is that, due to cultural issues, girls are likely to feel more comfortable expressing their feelings and admitting to their fears [42]. However, we did not find differences in fear scores between age groups, while a decrease in dental fear scores as age increased was demonstrated in many previous studies, both in cross-sectional studies [5,6,16,17] and longitudinal studies [50]. The authors of these studies suggest that the decrease in dental fear over time may be associated with an increase in general competence as the child grows and with the maturation of cognitive control of the child’s personality [1]. Some previous studies have also reported that age does not affect dental fear [51,52], or, conversely, increases dental fear at an older age [53].

Our results demonstrated that adolescents who had experienced caries were more likely to report higher dental fear scores than were caries-free adolescents, while in children, this was not the case. Regarding this association, only studies in children under 15 years of age can be found in the literature. Some of these studies confirmed the association between dental fear and caries experience [53,54,55], while others did not [56,57]. Moreover, there are studies that claim that dental fear could modify the association between dental caries and dental pain [58]. Among adolescents, because of the paucity of data, we could not compare the results of this testing with the findings of other studies.

Another dental characteristic that we studied was a self-reported rating of malocclusion. Testing has shown that it has no significant relationship with dental fear scores. Our previous study found that malocclusion, as well as caries experience, negatively affects OHRQoL [59]. The results of the present study show that a worse level of OHRQoL is associated with higher dental fear scores. This finding is in line with the results of studies by Merdad et al. (2017) [7] and Luoto et al. (2009) [8], where dental fear was a strong and significant predictor of poor OHRQoL in 11–14-year-old children. The recent meta-analysis by Alharbi et al. (2021) confirmed an overall minor association between child dental anxiety and OHRQoL [25]. Thus, it can be assumed that caries experience and malocclusion is related to dental fear not directly but through the mediator OHRQoL. A similar assumption can be made also regarding overall life satisfaction.

Researchers believe that psychological factors play an important role in the aetiology of some children’s dental fear and continue to affect individuals into adulthood [60]. There were not many psychological factors considered in our study, but self-esteem was one of them. It was revealed that poor self-esteem was associated with dental fear in both boys and girls, but more significantly in the older (17–18 years) adolescent group. A limited association between dental fear and self-esteem has been described in the study by Locker (2003) [61]. In a meta-analysis by Alharbi et al. (2021) [25], only one investigation was found, by Cinar et al. (2007) [62], in which the evidence of a minor negative association between child dental fear and self-esteem was also limited.

Dental fear appeared to be discriminated by socioeconomic factors [24,26,27,42]. In the present study, children and adolescents from rural areas (significantly in girls), living in unaffluent families (significantly in girls), and who had mothers with a low education level (significantly in 15–16-year-olds) were more likely to have dental fear. This finding can be attributed to the fact that participants from rural areas have less access to dental care in general, or the fact that children with unaffluent families have less access to better-quality dental care. In addition, mothers with a low education level might not perceive the need to seek early dental care for the prevention and treatment of dental problems in their children. The level of education of the father in this relationship was not consistent: it either had no significant association with dental fear, or a low education level was significantly associated with a lower dental fear score in children aged 11–13 years. Our findings on the association between parental education and child dental fear were in contrast with the findings from the study by Rantavuori et al. (2004), which reported that among 15-year-old Finns, if the mother had a higher level of education, the child was more likely to be afraid; however, if the father had a higher level of education, the child was less likely to be afraid [53].

Analysis of parental reports allowed us a deeper understanding of the antecedents of child dental fear. Initially, it has been verified that being older at their first visit to the dentist—for example, aged 6 years or more—increases the child’s likelihood of developing dental fear. This fact was confirmed only for the group of younger 11–14-year-olds but not for older participants, as their parents may have had difficulty remembering their first dental visit. The American Academy of Pediatric Dentistry (2014) encourages children to attend their first dental visit when their first tooth erupts or when they turn one year old, whichever comes first [63]. It is also recommended that the first visit to the dentist should be neutral (such as oral examination and prophylaxis), as children who receive invasive treatment during their first dental visit are more fearful [64]. The earlier their dental visits start, the sooner problems including dental fear can be addressed. Previous studies have confirmed this assumption, showing that children who had significantly higher scores of dental fear were those who had not visited the dentist at an early age or who had never been to the dentist [42,56]. The data provided by the parents in our study were also in agreement with these findings, as they denied the fact that an early visit to the dentist may be a precursor to dental fear. According to the parental opinion, it seems that a child’s dental fear could originate due to one or more of the following reasons: toothache that occurred during the treatment session, inappropriate dentists’ actions, high sensitivity of the child and poor psychological readiness for a visit to the dentist. Results similar to these were obtained in other studies as well [53,54,55].

Taking into account the high percentage of schoolchildren who reported high scores of dental fear, the close relationship of dental fear with untreated dental caries and the negative impact of dental fear on OHRQoL, considerable attention should be paid to the prevention of dental fear. Therefore, school-based health policies, paediatric dentists and parents should be encouraged to focus on the psychosocial factors associated with the fear of dental treatment because most of them can be prevented. There are a number of evidence-supported techniques that could be adapted to meet the needs of dentally anxious children and adolescents [3].

### Strengths and Limitations

Among the strengths of the current study, the major strength is the external data validity, which was ensured by the use of a large, randomly selected sample of the population and the use of valid measures; this allowed us to compare the current findings with previous studies. Another strength is that the data were collected by independent interviewing of students and their parents (students did not know their parents’ answers when answering the questionnaire). The comparison of respondents’ answers regarding their child’s dental fear showed good agreement that reaffirmed the validity of the data. The study provided important information on dental fear among adolescents in Lithuania.

It is also important to recognise some limitations in our study. First, the representativeness of our sample in terms of the gender and age of participants could be questioned. However, the groups of participants were reasonably balanced in the sample, and the prevalence of dental fear among participants was estimated in groups of appropriate sample size, which was within the range that has been reported in previous studies among children and adolescents [1]. Second, the data used in this study were collected by self-reported questionnaires. Respondents may have underestimated or, on the contrary, overestimated some of their behaviours, causing a desirability bias. For some questions, the adolescents may also have been unwilling to answer, causing a non-response bias. In addition, the questions answered by the parents were about the child’s early childhood; this could have led to memory bias. Third, this study was cross-sectional in nature. Therefore, no temporal relationship can be established and its findings can only suggest associations rather than causation [65]. Nevertheless, a cross-sectional study is important for its usefulness in searching for risk factors and in the planning of healthcare policies [66]. Finally, the sample of this study was drawn from children and adolescents in Lithuania. Therefore, the findings of this study cannot be directly extrapolated to other populations, although some useful implications can be drawn, especially for populations of similar cultures and social contexts.

Despite these limitations, we believe that the results of our study provide the analytical framework needed to better understand the problem of dental fear and develop an appropriate approach for its prevention among children and adolescents.

## 5. Conclusions

This study confirmed that dental fear among children and adolescents aged 13–18 years in Lithuania was characterised by a relatively high severity and prevalence compared to other studies. Girls reported greater dental fear scores than boys but the level of dental fear did not depend on the age. Higher levels of dental fear were significantly associated with untreated caries experience, a delay in the first visit to the dentist, low self-esteem, low oral health-related quality of life, low overall life satisfaction and low family affluence. It was also suggested that a child’s dental fear may be caused by a previous toothache, inappropriate dentists’ actions, high sensitivity or poor child’s psychological readiness for treatment.

From a public health perspective, the findings indicate that considerable attention should be given to the prevention of dental fear, which has a close relationship with untreated caries prevalence and has a negative impact on OHRQoL. Therefore, school-based health policies, paediatric dentists and parents should be encouraged to focus on the psychosocial factors associated with the fear of dental treatment because most of them can be prevented.

## Figures and Tables

**Table 1:** Distribution, n (%), of the sample, by gender and three age groups.

Gender	Age Group			Total
	11–14 Years	15–16 Years	17–18 Years	
Boys	128 (8.1%)	313 (19.7%)	222 (14.0%)	663 (41.7%)
Girls	179 (11.3%)	407 (25.6%)	341 (21.4%)	927 (58.3%)
Total	307 (19.3%)	720 (45.3%)	563 (35.4%)	1590 (100%)

**Table 2:** Sample characteristics reported by the students (*n* = 1590).

Characteristic	*n* (%) of Respondents	
Urban/rural residence:		
urban	1087	(68.5)
rural	500	(31.5)
missing	3	
Family affluence:		
high	794	(51.1)
medium	573	(36.9)
low	186	(12.0)
missing	37	
Self-reported general health status:		
good health	1356	(85.3)
less good health	233	(14.7)
missing	1	
Self-reported rating of caries experience:		
not at all	1043	(65.8)
treatment is needed	542	(34.2)
missing	5	
Self-reported rating of malocclusion:		
no anomalies	683	(43.1)
anomalies were observed	902	(56.9)
missing	5	
Teeth brushing:		
regular	1008	(63.4)
irregular	582	(36.6)
missing	0	
Self-esteem:		
high	787	(51.0)
low	755	(49.0)
missing	48	
Overall life satisfaction:		
high	994	(62.7)
low	591	(37.3)
missing	5	
Oral health-related quality of life:		
high	985	(61.9)
low	605	(38.1)
missing	0	

**Table 3:** Sample characteristics reported by the students’ parents (*n* = 1399).

Characteristic	No. (%) of Respondents	
Father’s education:		
lower	402	(43.9)
higher	514	(56.1)
missing	483	
Mother’s education:		
lower	374	(33.3)
higher	748	(66.7)
missing	277	
Age at the first visit to the dentist:		
up to 6 years	636	(54.2)
6 years or older	537	(45.8)
missing	226	
Parental opinion about child’s dental fear:		
has a dental fear	454	(32.6)
earlier had a dental fear	277	(19.9)
no dental fear	660	(47.4)
missing	8	
Dental fear was caused by the toothache:		
no	1286	(92.5)
yes	105	(7.5)
missing	8	
Dental fear was caused by dentist’s actions:		
no	1256	(90.3)
yes	135	(9.7)
missing	8	
Dental fear was caused by too young a child:		
no	1126	(80.9)
yes	265	(19.1)
missing	8	
Dental fear was caused by high child’s sensitivity:		
no	1168	(83.9)
yes	224	(16.1)
missing	7	
Fear was caused by poor psychological readiness:		
no	1317	(94.7)
yes	74	(5.3)
missing	8	

**Table 4:** Association of child and adolescent dental fear with gender, age, self-reported health status and tooth brushing habits.

Variable	Category	Relative Ratio of the Fear Score Means (95% CI) and *p*-Value					
		Total ^a^	Gender ^b^		Age Group ^c^		
			Boys	Girls	11–14 Years	15–16 Years	17–18 Years
Gender	Girls	1.19 (1.12–1.27) ***p* < 0.001**			1.19 (1.05–1.35) ***p* = 0.006**	1.24 (1.13–1.36) ***p* < 0.001**	1.14 (1.02–1.27) ***p* = 0.023**
	Boys	1.00			1.00	1.00	1.00
Age group	17–18 years	1.03 (0.95–1.12) *p* = 0.490	1.06 (0.94–1.20) *p* = 0.328	1.01 (0.90–1.13) *p* = 0.839			
	15–16 years	1.05 (0.97–1.13) *p* = 0.240	1.02 (0.91–1.15) *p* = 0.691	1.06 (0.95–1.19) *p* = 0.264			
	11–14 years	1.00	1.00	1.00			
Self-reported general health status	Less good health	1.00 (0.92–1.09) *p* = 0.962	0.91 (0.78–1.05) *p* = 0.194	1.06 (0.95–1.17) *p* = 0.323	0.88 (0.72–1.07) *p* = 0.192	1.03 (0.90–1.17) *p* = 0.675	1.04 (0.90–1.20) *p* = 0.565
	Good health	1.00	1.00	1.00	1.00	1.00	1.00
Self-reported rating of caries experience	Treatment is needed	1.14 (1.07–1.22) ***p* < 0.001**	1.12 (1.02–1.24) ***p* = 0.024**	1.16 (1.07–1.26) ***p ≤* 0.001**	1.14 (0.97–1.26) *p* = 0.143	1.14 (1.03–1.26) ***p* = 0.009**	1.17 (1.06–1.31) ***p* = 0.003**
	No carries lesions	1.00	1.00	1.00	1.00	1.00	1.00
Self-reported rating of malocclusion	Anomalies were observed	1.01 (0.95–1.08) *p* = 0.708	0.96 (0.87–1.05) *p* = 0.354	1.06 (0.97–1.15) *p* = 0.196	1.10 (0.97–1.25) *p* = 0.140	0.95 (0.86–1.04) *p* = 0.242	1.04 (0.93–1.16) *p* = 0.474
	No anomalies	1.00	1.00	1.00	1.00	1.00	1.00
Tooth brushing	Irregular	1.04 (0.97–1.11) *p* = 0.285	1.03 (0.94–1.13) *p* = 0.530	1.04 (0.95–1.14) *p* = 0.383	1.08 (0.95–1.23) *p* = 0.264	1.02 (0.92–1.12) *p* = 0.756	1.03 (0.92–1.16) *p* = 0.600
	Regular	1.00	1.00	1.00	1.00	1.00	1.00

Notes: ^a^ adjusted for gender and age group; ^b^ adjusted for age group; ^c^ adjusted for gender. The *p*-values of <0.05 are in bold.

**Table 5:** Association of child and adolescent dental fear with self-esteem, oral health-related quality of life and overall life satisfaction.

Variable	Category	Relative Ratio of the Fear Score Means (95% CI) and *p*-Value					
		Total	Gender		Age Group		
			Boys	Girls	11–14 Years	15–16 Years	17–18 Years
Self-esteem	Low	1.10 (1.04–1.18) ***p* = 0.002**	1.11 (1.01–1.22) ***p* = 0.032**	1.10 (1.01–1.19) ***p* = 0.027**	1.09 (0.96–1.24) *p* = 0.179	1.05 (0.96–1.16) *p* = 0.289	1.18 (1.06–1.31) ***p* = 0.003**
	High	1.00	1.00	1.00	1.00	1.00	1.00
Oral health-related quality of life	Low	1.13 (1.07–1.21) ***p* < 0.001**	1.13 (1.03–1.25) ***p* = 0.013**	1.14 (1.05–1.23) ***p* = 0.002**	1.23 (1.08–1.39) ***p* = 0.002**	1.06 (0.96–1.17) *p* = 0.241	1.17 (1.05–1.30) ***p* = 0.004**
	High	1.00	1.00	1.00	1.00	1.00	1.00
Overall life satisfaction	Low	1.07 (1.01–1.14) ***p* = 0.036**	1.10 (0.99–1.21) *p* = 0.062	1.05 (0.97–1.14) *p* = 0.260	1.03 (0.91–1.18) *p* = 0.636	1.05 (0.96–1.16) *p* = 0.296	1.13 (1.01–1.24) ***p* = 0.042**
	High	1.00	1.00	1.00	1.00	1.00	1.00

Notes: The *p*-values of <0.05 are in bold.

**Table 6:** Association of child and adolescent dental fear with several social factors.

Variable	Category	Relative Ratio of the Fear Score Means (95% CI) and *p*-Value					
		Total	Gender		Age Group		
			Boys	Girls	11–14 Years	15–16 Years	17–18 Years
Urban/rural residence	Rural	1.08 (1.01–1.15) ***p* = 0.026**	1.06 (0.95–1.16) *p* = 0.301	1.09 (1.01–1.19) ***p* = 0.040**	1.01 (0.88–1.16) *p* = 0.887	1.09 (0.99–1.20) *p* = 0.085	1.07 (0.96–1.20) *p* = 0.208
	Urban	1.00	1.00	1.00	1.00	1.00	1.00
Family affluence	Low	1.11 (1.01–1.22) ***p* = 0.042**	1.03 (0.87–1.21) *p* = 0.744	1.15 (1.02–1.30) ***p* = 0.021**	1.12 (0.91–1.37) *p* = 0.303	1.04 (0.90–1.20) *p* = 0.607	1.24 (1.05–1.46) ***p* = 0.011**
	Middle	1.04 (0.98–1.11) *p* = 0.219	1.07 (0.96–1.19) *p* = 0.204	1.03 (0.94–1.12) *p* = 0.557	1.08 (0.95–1.24) *p* = 0.252	1.02 (0.92–1.12) *p* = 0.780	1.08 (0.96–1.21) *p* = 0.203
	High	1.00	1.00	1.00	1.00	1.00	1.00
Father’s education	Lower	1.00 (0.93–1.09) *p* = 0.920	1.00 (0.87–1.14) *p* = 0.997	0.99 (0.89–1.09) *p* = 0.986	0.80 (0.65–0.97) ***p* = 0.020**	1.09 (0.97–1.22) *p* = 0.164	1.00 (0.86–1.14) *p* = 0.925
	Higher	1.00	1.00	1.00	1.00	1.00	1.00
Mother’s education	Lower	1.08 (1.01–1.17) ***p* = 0.041**	1.07 (0.95–1.21) *p* = 0.289	1.08 (0.98–1.19) *p* = 0.107	0.96 (0.81–1.13) *p* = 0.599	1.13 (1.01–1.20) ***p* = 0.033**	1.11 (0.97–1.26) *p* = 0.126
	Higher	1.00	1.00	1.00	1.00	1.00	1.00

Notes: The *p*-values of < 0.05 are in bold.

**Table 7:** Association of child and adolescent dental fear with variables reported by parents.

Variable	Category	Relative Ratio of the Fear Score Means (95% CI) and *p*-Value					
		Total	Gender		Age Group		
			Boys	Girls	11–14 Years	15–16 Years	17–18 Years
Parental opinion about child’s dental fear	Have a dental fear	1.59 (1.48–1.71) ***p* < 0.001**	1.53 (1.37–1.72) ***p* < 0.001**	1.63 (1.49–1.78) ***p* < 0.001**	1.56 (1.35–1.82) ***p* < 0.001**	1.66 (1.49–1.85) ***p* < 0.001**	1.52 (1.34–1.72) ***p* < 0.001**
	Earlier had a dental fear	1.08 (0.99–1.19) *p* = 0.094	1.06 (0.92–1.24) *p* = 0.419	1.10 (0.98–1.24) *p* = 0.110	1.03 (0.84–1.27) *p* = 0.751	1.11 (0.97–1.28) *p* = 0.133	1.09 (0.92–1.28) *p* = 0.313
	No dental fear	1.00	1.00	1.00	1.00	1.00	1.00
Age at the first visit to the dentist	6 years or older	1.08 (1.01–1.16) ***p* = 0.036**	1.12 (0.99–1.26) *p* = 0.065	1.04 (0.95–1.14) *p* = 0.353	1.17 (1.01–1.36) ***p* = 0.040**	1.04 (0.94–1.16) *p* = 0.454	1.04 (0.92–1.16) *p* = 0.506
	Up to 6 years	1.00	1.00	1.00	1.00	1.00	1.00
Fear was caused by toothache	Yes	1.18 (1.06–1.32) ***p* = 0.003**	1.16 (0.98–1.36) *p* = 0.078	1.21 (1.05–1.41) ***p* = 0.011**	1.54 (1.22–1.95) ***p* < 0.001**	1.04 (0.88–1.22) *p* = 0.653	1.24 (1.02–1.52) ***p* = 0.032**
	No	1.00	1.00	1.00	1.00	1.00	1.00
Fear was caused by the dentist’s actions	Yes	1.09 (1.04–1.15) ***p* = 0.001**	0.97 (0.86–1.08) *p* = 0.529	1.13 (1.07–1.20) ***p* < 0.001**	1.01 (0.90–1.13) *p* = 0.914	1.09 (1.01–1.17) ***p* = 0.031**	1.16 (1.06–1.26) ***p* = 0.001**
	No	1.00	1.00	1.00	1.00	1.00	1.00
Fear was caused by too young child	Yes	1.01 (0.98–1.04) *p* = 0.448	1.02 (0.97–1.06) *p* = 0.498	1.01 (0.97–1.05) *p* = 0.634	1.04 (0.98–1.11) *p* = 0.181	1.03 (0.98–1.07) *p* = 0.242	0.98 (0.93–1.03) *p* = 0.354
	No	1.00	1.00	1.00	1.00	1.00	1.00
Fear was caused by high child’s sensitivity	Yes	1.08 (1.06–1.10) ***p* < 0.001**	1.05 (1.02–1.09) ***p* = 0.005**	1.09 (1.06–1.12) ***p* < 0.001**	1.06 (1.02–1.11) ***p* = 0.010**	1.08 (1.05–1.11) ***p* < 0.001**	1.08 (1.04–1.12) ***p* < 0.001**
	No	1.00	1.00	1.00	1.00	1.00	1.00
Fear was caused by poor psychological readiness	Yes	1.05 (1.02–1.08) ***p* < 0.001**	1.05 (1.01–1.10) ***p* = 0.024**	1.05 (1.02–1.09) ***p* = 0.003**	1.04 (0.96–1.13) *p* = 0.374	1.06 (1.03–1.10) ***p* = 0.001**	1.04 (0.99–1.08) *p* = 0.108
	No	1.00	1.00	1.00	1.00	1.00	1.00

Notes: The *p*-values of <0.05 are in bold.

## Data Availability

The dataset supporting the conclusions of this article is available from the corresponding author on reasonable request.
